# Ethical and regulatory implications of the COVID-19 pandemic for the medical devices industry and its representatives

**DOI:** 10.1186/s12910-022-00771-2

**Published:** 2022-03-23

**Authors:** Brette Blakely, Wendy Rogers, Jane Johnson, Quinn Grundy, Katrina Hutchison, Robyn Clay-Williams, Bernadette Richards, Guy Maddern

**Affiliations:** 1 Department of Philosophy, Macquarie University, North Ryde, NSW Australia; 2 The Macquarie University Research Centre for Agency, Values, and Ethics (CAVE), Macquarie University, North Ryde, NSW Australia; 3 School of Medicine, Macquarie University, North Ryde, NSW Australia; 4 Lawrence S. Bloomberg Faculty of Nursing, University of Toronto, Toronto, ON Canada; 5 Australian Institute of Health Innovation, Macquarie University, North Ryde, NSW Australia; 6 Adelaide Law School, The University of Adelaide, Adelaide, SA Australia; 7 Future Health Technologies of the Singapore-ETH Centre at CREATE, Singapore, Singapore; 8 Discipline of Surgery, The University of Adelaide, Adelaide, SA Australia; 9 The Queen Elizabeth Hospital, Woodville South, SA Australia

**Keywords:** Device representatives, Device regulation, COVID-19, Ethics, Crisis

## Abstract

The development and deployment of medical devices, along with most areas of healthcare, has been significantly impacted by the COVID-19 pandemic. This has had variable ethical implications, two of which we will focus on here. First, medical device regulations have been rapidly amended to expedite approvals of devices ranging from face masks to ventilators. Although some regulators have issued cessation dates, there is inadequate discussion of triggers for exiting these crisis standards, and evidence that this may not be feasible. Given the relatively low evidence standards currently required for regulatory approval of devices, this further indefinite reduction in standards raises serious ethical issues. Second, the pandemic has disrupted the usual operations of device representatives in hospitals, providing an opportunity to examine and refine this potentially ethically problematic practice. In this paper we explain and critically analyse the ethical implications of these two pandemic-related impacts on medical devices and propose suggestions for their management. These include an endpoint for pandemic-related adjustments to device regulation or a mechanism for continued refinement over time, together with a review of device research conducted under crisis conditions, support for the removal and replacement of emergency approved devices, and a review of device representative credentialling.

## Background

The medical device industry plays a central role in the provision of healthcare. Globally, the industry is highly competitive and is expected to generate US$432.6 billion by 2025 [1]. However, as with other areas of healthcare and society [2], the COVID-19 pandemic has shone a harsh light on vulnerabilities in device regulation and deployment. The critical need for ventilators and in some cases, widespread recalls of medical supplies [3] are examples of how the pandemic has highlighted the reliance of healthcare on the timely provision of safe and effective medical devices.

Typically, in the regulation of medical devices certain key practical and ethical principles are used in setting standards. Specifically, the principle of harm minimisation stipulates the importance of avoiding harm to individual patients, necessitating rigorous safety testing and monitoring, followed by swift recalls as required. However, in response to the COVID-19 pandemic, medical device regulators around the world have issued amended or expanded approvals in order to meet the unprecedented demand for devices ranging from face masks to ventilators [4–9].

Internationally, policy amendments have clearly relaxed usual safety precautions. In the US, the Secretary of the Department of Health and Human Services (HHS) authorised emergency use of: “…in vitro diagnostics for the detection and/or diagnosis of COVID-19 (February 4, 2020), personal respiratory protective devices (March 2, 2020), and other medical devices, including alternative products used as medical devices (March 24, 2020), for use during the COVID-19 outbreak…” [4]. Emergency Use Authorisations (EUAs) can be issued for devices which “may be effective”, which is a lower threshold than normally required [10].

In Europe, special rules for Personal Protective Equipment (PPE) and medical devices needed to respond to the “current health crisis” were outlined in a European Commission (EC) document of 16th March 2020 [5, 8] and include provisions for alignment of expectations across EU member states. Recommendation 1 calls for ensuring the supply of PPE and devices which provide “adequate protection” by encouraging any relevant agencies or bodies to “employ all the measures at their disposal” (L 79 I/4 5). Perhaps more significantly member states are permitted to authorize marketing of individual devices that have not yet been assessed for conformity where these devices are urgently required due to disruption of usual supply chains of approved alternatives, or there is no approved alternative device [11]. The EC also postponed (from May 2020 until May 2021) the implementation of updated device regulation intended to close loopholes in device approval processes [6], such as those associated with high profile device scandals, including that of trans-vaginal mesh [12].

In Australia, the Therapeutic Goods Administration (TGA) response to COVID-19 includes procedures for rapid assessment of a range of medical devices used in the treatment of the disease [7]. These include ventilators, point-of-care testing kits and PPE. *The Therapeutic Goods Act* 1989 provides an exemption for medical devices that might be urgently needed for public health emergencies such as COVID-19 but that are not already registered on the Australian Register of Therapeutic Goods (ARTG). This exemption remained in place for face masks (and related PPE) and ventilators until 31 Jan 2021 [8, 9]. The hasty acquisition of certain items has already resulted in the sale and purchase of products that failed to meet regulatory standards [3].

As the hands-on experts in these rapidly evolving products, medical device representatives previously maintained a strong and pervasive presence in hospitals [13, 14]. Their role included supporting the introduction and use of medical devices by clinicians in a variety of clinical settings such as operating theatres, patient care units, and cardiac catheterisation labs [15]. The extensive presence of medical device representatives in hospitals, and more specifically operating theatres, triggered significant ethical concerns about the role of commercial incentives in clinical decision-making and patient privacy [14]. However, pandemic measures such as bans on visitors that included sales representatives promoting their products [16] and the sudden cancellation of elective surgeries, led to the much-reduced presence of device representatives in hospitals, necessitating significant adaptation within the device industry.

These COVID-19 pandemic disruptions to the medical device industry have as yet unknown long-term consequences. In this analysis we take an ethics perspective to examine the influence of the COVID-19 pandemic on the roles of device companies and representatives. First, we examine whether the consequences of adapting regulatory systems in response to emergency conditions may be more far reaching and permanent than intended, putting patients at significant risk of adverse events due to device-related failures. Second, we consider how other adaptations regarding the role and regulation of device representatives may indeed be possible and perhaps should be made permanent.

## Main text

### How a crisis changes priorities: COVID-19 modifications to device regulation

The declaration of a crisis (like the WHO announcement of COVID-19 as a pandemic on 11 March 2020), is not just an exercise in semantics. Disaster labels allow for special use of administrative and other powers. Historically, emergency powers are designed to be ‘conservative’ in that they are limited to the duration of the emergency situation, after which there should be a return to the previous state which the emergency threatened [17]. The emergency declaration allows for measures which would not, normally, be tolerated: “[t]he entire point of declaring a state of emergency is to enable an exceptional response that is not permissible during a state of ‘normalcy’…” [18] p 9. In the public health context, one of the main underlying concerns is that the emergency will result in a demand for hospital based healthcare “that exceeds or threatens to exceed the healthcare organization’s surge capacity” [19]. An important element which allows for and sanctions these responses is a shift in ethical reasoning which underpins decision making. In the case of the COVID-19 pandemic, the emergency response included changes to the regulations governing practices such as device approvals.

The introduction of medical devices creates risk of novel harms from poor design, faulty manufacture, or incorrect use. To outweigh this risk and minimize harm, new technologies must be shown to provide equal or greater benefit than existing devices or other alternatives such as not intervening. Given the health impacts of the pandemic, and a surge in demand for critical supplies and equipment, regulators shifted to prioritise speed of approvals over scrutiny of evidence and safety data (the efficiency-thoroughness trade-off (ETTO)) [20]. Regulators made changes which lowered the evidence requirements that could otherwise impede availability of essential medical supplies and devices.

The premium placed on enhanced supply reveals a shift in ethical evaluations. Clearly this expedience may be necessary as continuing ‘business-as-usual’ may result in unacceptable risks, such as delayed access to and inefficient allocation of resources during the pandemic, with attendant increased morbidity and mortality. However, the haste to secure supplies of essential devices was at times counter-productive. For example, a different threshold was adopted for establishing efficacy of COVID-19 test kits in early 2020, compared to prevailing standards for diagnostic tests. This led to unknown and potentially increased risks to both patients and the public health [21]. In the US, approval for some masks has been withdrawn and increased sampling and surveillance of shipments from China instigated [22]. In Australia, the TGA instituted an expedited recall pathway for this purpose (initiated in < 24 h), which can be triggered by reports or complaints regarding safety, quality, efficacy, performance or presentation [23]. As these examples show, the efficiency-thoroughness trade-off is a delicate balance. The aim of saving more lives may not be achieved if the rapid approvals lead to faulty devices.

### COVID-19 as an evolving crisis with lasting effects

A key feature of emergency declarations is the temporary nature of the exceptional powers with the assumption of a return to normal. However, in the case of device regulation during the pandemic, both ending the use of exceptional powers and the ongoing after effects may be difficult to manage. The ethical decision making which was justified during the crisis may inadvertently be sustained due to at least two factors.

First, the endpoint of a pandemic is not as well-defined as some emergency events like a flood or enemy invasion. The COVID-19 pandemic is a dynamic and evolving situation with unknown long-term effects for individuals and populations [24]. Therefore, determining appropriate end dates or ‘sunset clauses’ may prove difficult. Even if the need for ventilators or PPE decreases as infection rates come under control, there may be an urgent need for sufficient devices, such as ones for cardiac support, for patients with ongoing COVID-19-related health problems. This means: “…the crisis of COVID-19 will not abate but will instead shift to a new de novo incidence of heart failure and other chronic cardiovascular complications” [25]. The ethical imperative to meet pandemic-related urgent healthcare needs is, therefore, unlikely to suddenly end, meaning an ongoing and delicate balance will need to be maintained for years, if not decades.

Second, once devices approved under pandemic regulations are in hospitals, it is unlikely that they will be removed from circulation at the end of the pandemic. Approved devices have included ventilators, infusion pumps, blood purification, renal replacement and hemodialysis devices, and left ventricular support systems [4]. Even during the initial crisis, the fact that emergency approval might suddenly be revoked and therefore required almost daily checking was cause for concern. These risks caused ECRI (founded as Emergency Care Research Institute) to rank “Complexity of Managing Medical Devices with COVID-19 Emergency Use Authorization” first of the Top 10 Health Technology Hazards of 2021 [26]. Furthermore, without strong evidence of fault or complaint, it is difficult to recall devices that enter practice even if there are more effective or cheaper alternatives. Exacerbating this problem is the fact that hospitals, driven by scarcity, were forced into quickly investing at potentially higher than normal prices, contributing to increased health care costs.

Concerns over the influence of the device industry on treatment and purchasing decisions in healthcare are not new. While previous scrutiny has centred on the relationships between physicians and device manufacturers [13, 15], concern has now shifted to how the device industry itself might operate in an environment of loosened restrictions and surges in demand. Worries over inflated prices with new middle men brokering deals [27] and subpar products have led to allegations of a “Wild West” market [28]. Thus hospitals, desperate to secure limited stocks have had to stretch funds and are unlikely to dispense with or be able to replace devices acquired during the crisis. The ethical commitment of the healthcare system to minimise harms is potentially compromised by reliance on a device industry that does not have the same obligations, and indeed has entirely different incentives. Device representatives often work on a commission basis with instructions to encourage use of devices that are newer and more expensive, rather than safer or more effective [13]. Although device companies clearly need to meet regulatory requirements, they have no obligations to patients or providers to keep down costs, enhance efficacy or increase device lifespan.

### Recommendations to reinstate prior ethical standards

Together these issues have the capacity to introduce risks to patient safety that may be difficult to dislodge once the pandemic is over. This is ethically problematic because the claimed justification for crisis standards will no longer prevail, but the risks associated with the emergency measures will remain. Several steps should be taken to address these concerns:

- instigate annual review of the types of devices that fall under modified regulations, in order to limit the number and scope of devices which are approved under emergency standards and ensure that they closely align with changing healthcare needs as the pandemic evolves;
- retrospectively require additional evidence of safety and efficacy for all devices and device related research produced under emergency conditions, to mitigate the longer-term harmful impacts of any hastened processes;
- instigate support mechanisms for hospitals to ensure removal or replacement of devices once their emergency approvals have ceased.

As recommendations like these are not within the purview of any particular regulatory body, in the first instance responsibility could be assumed by the relevant professional bodies, devolving to local hospital boards once appropriate guidelines are established.

### COVID-19 exacerbation of existing issues in device representative practices

As well as raising new challenges, pandemic conditions also shed light on issues raised by the under-regulation of the activities of device representatives in clinical settings. Ethical concerns over the presence of device representatives in hospitals and operating theatres have typically involved concerns about physicians’ conflict of interest and patient privacy [14]. Unlike pharmaceutical representatives, device representatives are allowed into clinical spaces during consultations and sterile procedures, at the request of the clinician, and have access to highly sensitive data [29]. Clinicians report reliance upon device representatives for technical advice and are sometimes unable or unwilling to perform certain procedures in their absence [15]. In other cases, hospitals engage device representatives to provide device-related education and support in the procurement contract, which is perceived as a value-add and competitive advantage [30]. In all cases, the outsourcing of technical education and support to representatives operating in a sales capacity for for-profit manufacturers raises questions about accountability, role definition, and the influence of commercial incentives [15].

In anticipation of a surge of COVID-19-related hospitalisations, many health systems sought to ensure capacity by cancelling elective surgeries, reducing hospital attendance by device representatives. Cancelling surgeries also served to reduce the risk of COVID-19 exposure and transmission by decreasing the overall number of people in hospitals. As many device representatives visit multiple hospitals in the course of their work, they are an occupational group at high risk of spreading infection to various sites should they be infected. Though recognition of device representatives as potential infection risks in clinical settings predate the pandemic [29], these new policies provoke further questions about the necessity of having sales representatives in hospital settings at all, and if permitted, what policies and safeguards should apply.

Pre-pandemic, health systems around the world have tended to take a risk management approach to the presence of device representatives in clinical settings. Regulatory bodies such as the Joint Commission (United States), publish standards related to identification, infection control, and privacy. In response, industry has advocated for uniform “vendor credentialing” standards to facilitate representative access to hospitals [31]. In the United States, representatives must pay a fee to register with a vendor credentialing company which compiles, verifies, and documents representatives’ immunisations, police checks, and completion of training modules like sterile practices and infection control [16]. As health systems re-open and re-start elective surgeries, it is timely to ask whether an industry self-regulatory system and industry standards for infection control are adequate or require revision and/or oversight. And, given the heightened risks of infection, the physical presence of sales representatives in clinical settings requires robust justification.

### Opportunities and limitations presented by pandemic related adaptations for device representative interactions

During the current pandemic, device representatives’ access to healthcare facilities has been heavily restricted and they were required to work from home by their companies, who sought to protect their workforce and others [32]. In response, sales representatives have sought new ways to communicate remotely with health professionals. Some companies now offer enhanced video resources and webinars [33], whilst others are expanding their “tele-detailing” or virtual meeting capabilities [34]. In some instances, tele-detailing was already standard due to inability to access specialist representatives, particularly for rarely used equipment. While adoption of “tele-detailing” has not been rapid or universal, it suggests a possible future trend for healthcare sales and support given the ongoing threat of COVID-19 and the likelihood of future pandemics [35].

Although a move away from the physical presence of device representatives in hospitals may seem to reduce practical issues such as infection risk, at the same time it exacerbates existing, and introduces novel, practical and ethical considerations, as there are significant regulatory gaps around digital health technologies [36]. As practises have evolved and device companies have gained increased access to identifiable and de-identified patient data, regulatory frameworks have failed to evolve at the same rate and incorporate the relevant ethical considerations. “Tele-reps” may reduce infection risks, however by expanding the amount of digital data created they pose additional challenges including: managing data security given the value of any health professional user data collected for marketing and sales; protecting patient privacy given that tele-rep consultations may include cameras operating in clinical spaces; and regulation of promotion given that all information shared through the app likely has promotional intent or consequence [37]. This increase in digital data introduces new types of risk associated with maintaining digital patient privacy. It may also further reduce transparency regarding the ‘presence’ and influence of device representatives in clinical spaces by making it less obvious and therefore potentially more difficult to track and manage conflicts of interest. Conversely, by providing remote and more central healthcare settings with equivalent support, the move towards tele-reps may result in more just access to this expertise.

One possible response to the challenges articulated above would be a radical rethinking of the system so that hospital employees are trained to do essential hospital based components of the work currently done by device representatives. This could potentially address both the ethical challenges associated with conflicts of interest arising from relationships between health professionals and device representatives [13, 14], and the COVID-19 related risks of device representatives in sterile settings and traveling between hospitals. However, this would be a significant departure from current practice, posing new challenges and giving rise to potential new risks of harm. It may not be possible for in-house staff to develop appropriate expertise across the wide range of devices in use at each institution to adequately stand in for device representatives in all procedures. Such a change would necessitate further integration of health technology assessment into day-to-day purchasing and clinical decision-making. Furthermore, with increasing budget pressures, it is unlikely that staff could be paid to undertake the extra training and additional work required. However, this approach may be more cost-effective in the long term given the many costs associated with the presence of device representatives [15].

## Conclusions

The COVID-19 pandemic has highlighted weaknesses in, and thereby provided an opportunity to critically re-examine, device regulatory standards, including approvals and the activities of device representatives. Due to the evolving nature of the pandemic and the lasting effects of allowing speedily approved and over-priced devices into the market, the ethical implications may be long lasting. Globally, the changes made to device regulation in response to the COVID-19 pandemic have the potential to inadvertently threaten patient safety long after expedited approvals cease, assuming they ever do. We need clear and dynamic re-evaluation and ‘stopping points’ for relaxed COVID-19 regulation along with post-pandemic reviews of device approvals to ensure removal of devices whose approvals have lapsed. In addition, measures for improved transparency such as the European Standards should be made freely available [38]. Without a co-ordinated international response, lowered standards may become the ‘new normal’, with adverse effects for patients.

The effect of the pandemic on elective surgeries and visiting rights in hospitals has also provided an opportunity to re-examine the role of device representatives in clinical settings. The need to essentially ban device representatives from hospitals demonstrates that current credentialing is insufficient to mitigate the infection risk posed to and by device representatives in clinical settings. While a move to remote or virtual platforms may reduce infection risk, fundamental questions remain about outsourcing such expertise and the associated patient safety issues.

## Data Availability

Not applicable.

## Abbreviations

ARTG: Australian Register of Therapeutic Goods
ECRI: Founded as Emergency Care Research Institute now ECRI
EC: European Commission
EUA: Emergency Use Authorization
HHS: Department of Health and Human Services
PPE: Personal Protective Equipment
TGA: Therapeutic Goods Administration
